# TMVR after TA-TAVR: a re-redo surgery—case report

**DOI:** 10.3389/fcvm.2024.1373840

**Published:** 2024-05-20

**Authors:** Nina Sophie Pommert, Thomas Puehler, Inga Voges, Stephanie Sellers, Georg Lutter

**Affiliations:** ^1^ Department of Cardiac Surgery, University Medical Center Schleswig-Holstein, Campus Kiel, Kiel, Germany; ^2^ DZHK (German Centre for Cardiovascular Research), Partner Site Hamburg/Kiel/Luebeck, Kiel, Germany; ^3^ Department of Cardiac Surgery, University Medical Center Schleswig-Holstein, Campus Luebeck, Luebeck, Germany; ^4^ Department of Congenital Heart Disease and Pediatric Cardiology, University Children’s Hospital Kiel, Kiel, Germany; ^5^ Centre for Cardiovascular Innovation, St Paul’s and Vancouver General Hospital, Vancouver, BC, Canada; ^6^ Cardiovascular Translational Laboratory, Providence Research & Centre for Heart Lung Innovation, Vancouver, BC, Canada; ^7^ Centre for Heart Valve Innovation, St. Paul’s Hospital, University of British Columbia, Vancouver, BC, Canada

**Keywords:** TAVR, TMVR, Tendyne, redo transapical access, case report

## Abstract

**Introduction:**

Transcatheter mitral valve replacement (TMVR) is a valuable treatment option in patients with severe mitral regurgitation. Prior transapical transcatheter aortic valve replacement (TA-TAVR) may complicate the procedure and is therefore considered a relative contraindication. In this case report, the authors describe the successful TMVR as a tertiary cardiac surgery and transapical redo procedure.

**Case Summary:**

An 83-year-old male patient, suffering from dyspnoea and angina, was diagnosed with severe mitral valve regurgitation (MR). He had already undergone cardiac surgery in the form of coronary artery bypass grafting at the age of 64 and TA-TAVR at 79 years. After a failed attempt at mitral valve transcatheter edge-to-edge repair, he opted for TMVR. Pre-TMVR computed tomography simulation was used to analyse possible interactions between the prostheses and to predict the neo-left ventricular outflow tract (neo-LVOT). The operation was carried out without complications. There was no bleeding and the LV function remained unchanged. On MRI, the valves were perfectly aligned without any signs of paravalvular leakage or LVOT obstruction. The patient was discharged seven days postoperatively. At the one-year follow up, there was no need for rehospitalisation and the patient had clinically improved (from NYHA IV to II). Echocardiography demonstrated a mean transvalvular gradient of under 5 mmHg and no residual MR.

**Conclusion:**

A redo transapical access for TMVR as a tertiary cardiac operation can be easily performed. Pre-operative CT suggested good alignment of the aortic and mitral valved stent which was confirmed postoperatively.

## Introduction

Transcatheter mitral valve replacement (TMVR) has become a valuable treatment option in patients with severe mitral regurgitation (MR). Previous aortic valve replacement is considered a relative contraindication for the procedure due to the risk of left ventricular outflow tract (LVOT) obstruction and interactions between the two prostheses and anchoring mechanism. Moreover, in transapical redo surgery, complications at the access site may occur. In this case, the authors describe the successful TMVR as a tertiary cardiac surgery using the Tendyne® system.

## Case presentation and diagnostic assessment

An 83-year-old male patient presented with dyspnoea and angina under cardiac decompensation (NYHA III). He had previously been treated with coronary artery bypass grafting for severe three-vessel disease at the age of 64 and transapical transcatheter aortic valve replacement (TA-TAVR, 29 mm S3) for aortic valve stenosis at 79 years. Coronary treatment was complemented by percutaneous coronary intervention and stenting of the PL branch, also at 79 years. Moreover, he was known to have arterial and pulmonary artery hypertension, chronic obstructive lung disease and peripheral artery disease with a history of bilateral femoropopliteal bypass surgery (Table 1). Before the start of the complaint, he lived at home independently.

**Table 1:** Patient timeline.

Timeline	
1999	Bilateral femoropopliteal bypass surgery
2003	Coronary artery bypass grafting (RIMA to LAD, SVG to PDA and PL)
09/2018	Cardiac decompensation NYHA IV
09/2018	PCI and stenting of PL branch
11/18	Transapical TAVR (29 mm S3)
06/22	Onset of angina and dyspnoea NYHA III-IV - Exclusion of relevant coronary stenoses - Diagnosis of severe MR
08/22	Failed TEER
09/22	TMVR (Tendyne 29 L)
09/22	Discharge home in good clinical condition
09/23	Follow-up at 12 months: good clinical condition, living independently at home, dyspnoea (NYHA II)

On admission, blood pressure was elevated (180/100 mmHg); heart rate (71 bpm) and room air oxygen saturation (98%) were normal. Pulmonary auscultation revealed reduced breathing sounds, in line with pulmonary oedema in the thoracic x-ray. The ECG showed a normal frequency sinus rhythm with left axis deviation and bifascicular block. Echocardiography revealed severe secondary mitral valve regurgitation (MR) with an effective regurgitation orifice area (EROA) of 29 mm^2^ with a vena contracta width of 7 mm, and a regurgitation volume of 50 ml. The left ventricular function was moderately impaired (ejection fraction 36%, left ventricular end diastolic volume 215 ml) and there was moderate tricuspid regurgitation. Laboratory analysis on admission revealed elevated of NT-proBNP (6,417 ng/L). Due to elevated troponin T with an increasing trend, coronary angiography was performed, excluding renewed coronary stenoses.

## Therapeutic intervention

The patient was discussed by a multidisciplinary heart team and initially primed for mitral valve transcatheter edge-to-edge repair, but the procedure was not successful due to strong tethering of the posterior leaflet. Considering the high-risk profile (STS-Score 7.5%, EuroScore II 35.4%) the patient opted for TMVR with the Tendyne® system.

**Figure 1:**
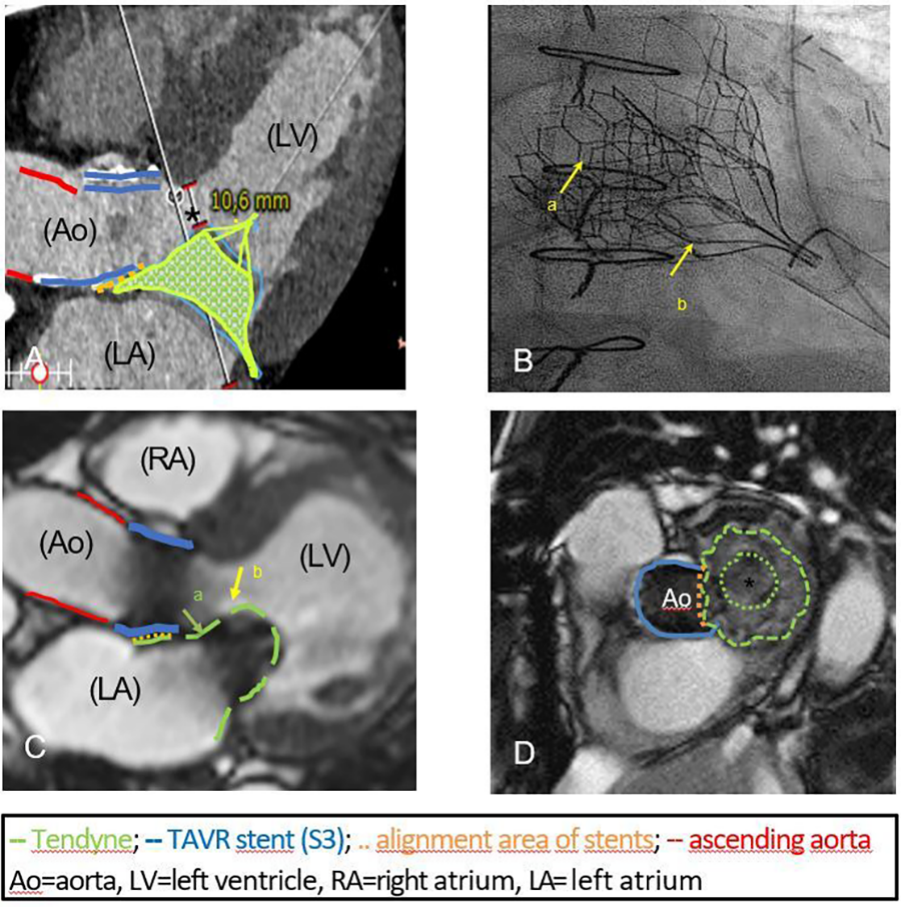
(**A**) Three-chamber view in CT simulation, predicting an end-diastolic neo-LVOT of 10.6 mm (*). The simulated alignment area of the aortic (29 mm S 3, blue line) and mitral prosthesis (green cage) is marked by the dotted orange line. The red line indicates the ascending aorta. (**B**) Fluoroscopy at the end of the procedure: 29 mm S 3 (arrow a) and the overlapping crown of the 29 mm Tendyne valve (low profile, arrow b) are fully deployed without interference. (**C**) Postprocedural MRI documenting the well-placed intra-annular position of Tendyne valve (arrow a) and its distal part targeting the anterior leaflet (hyperintense, arrow b, length 23.4 mm). The ascending aorta (red line), the cylinder of the S3 (blue line), the implanted Tendyne® stent (green line) and the overlapping area (dotted orange line) are highlighted. No LVOT-obstruction. (**D**) Optimal alignment (orange dotted line) of the S3 (blue line) and Tendyne prothesis (green line) on postoperative MRI. The circular inner frame of the Tendyne valve with porcine pericardial leaflets (*) is mounted on a self-expanding outer frame.

Pre-procedural computed tomography (CT) simulation was used to determine the ideal access and exclude interactions between the mitral and aortic valve prosthesis. Special attention was paid to the neo-LVOT, which was predicted to have 10.6 mm (Figure 1A).

TMVR was performed under echocardiographic and fluoroscopic guidance, using the previous anterolateral thoracotomy for transapical access. The partially pulmonary adhesion was carefully dissected from the thoracic wall and the cardiac apex, revealing the pledges of the previous transapical procedure. For pucturing of the left ventricle with optimal angulation to the mitral position, a site posterolateral to the previous access was chosen. After apical pre-dilation with a 21F sheath, a 26F sheath was placed, through which the implantation was carried out without complications. Fluoroscopy by the end of the procedure confirmed optimal valve positioning (Figure 1B). The patient was extubated in the operating room and was admitted to normal ward on the first postoperative day.

Postoperative magnetic resonance imaging (MRI) showed optimal positioning of the prosthesis in the mitral anulus without any sign of paravalvular leakage, which was confirmed by echocardiography (Figures 1C,D). LV function remained moderately impaired. As predicted, there was no obstruction of the neo-LVOT. Haemoglobin was stable postoperatively with no need for transfusions.

The patient was discharged home seven days postoperatively. He subsequently participated in a three-week inpatient cardiac rehabilitation programme. There were no postoperative complications according to the MVARC criteria and no further inpatient admission was necessary in the year that followed. Echocardiography excluded paravalvular leakage or relevant transvalvular gradients (< 5 mmHg). LV function was only lightly impaired. The patient continued to live independently at home with improved exercise capacity (NYHA II).

## Discussion

Multi-valvular disease in elderly, multimorbid patients is an important issue to deal with. With more than 1,700 procedures performed worldwide, TMVR with the Tendyne® system (Abbott) is an emerging treatment strategy for severe mitral valve regurgitation in high-risk patients.

As it is potentially fatal, LVOT obstruction is of major concern in patients with TMVR. Implantation of the prosthesis leads to the formation of the so called neo-LVOT, confined by the native anterior mitral leaflet fixed in an opened position, the valve stent and the septum (1). LVOT obstruction may lead to acute hemodynamic deterioration or chronic heart failure due to an increased left ventricular afterload (1, 2). As a consequence, pre-procedural computed tomography simulation and measurement of the predicted LVOT has gained in importance (1).

An existing aortic valve prosthesis may add complexity as it influences aortomitral angulation. Moreover, LV hypertrophy as a consequence of long-standing aortic valve stenosis may narrow the LVOT. We therefore relied on CT simulation in this complex case to visualise the planned implantation and ensure there was an adequate LVOT area.

The feasibility of TMVR in the presence of a surgical or transcatheter aortic valve prosthesis has previously been described previously (3, 4). Similarly, simultaneous transapical implantations of an aortic and mitral valve prosthesis has been reported (5). However, in both aortic and mitral valve surgery, little is known about transapical re-intervention. Despite the fear of apical fragility, individual reports describe good results of redo transapical TAVR within a week, three or seven years after the initial procedure (6–8). As in our case, postoperative adhesions were easily controllable and the apical tissue was rated unexpectedly normal (6). In the cases mentioned of redo transapical TAVR, the same apical access site was used. For optimal angulation in our patient, we had to use a new apical access site posterolateral of the previous position. Transapical access may cause myocardial damage and scarring. However, the existence of two adjacent accesses did not lead to restriction of the left ventricular function in our patient. He showed moderately impaired LV function in both pre- and postprocedural echocardiography and MRI (ejection fraction 36% pre- and 37% post-procedurally). There was even an improvement to a lightly impaired left ventricular function in the one-year follow up.

In conclusion, this case demonstrates that a redo transapical access for TMVR as a tertiary cardiac procedure can be easily performed with good clinical results. Accurate pre-operative screening is crucial.

## Data Availability

The original contributions presented in the study are included in the article/Supplementary Material, further inquiries can be directed to the corresponding author.
